# Segmentation of Brain MRI Using SOM-FCM-Based Method and 3D Statistical Descriptors

**DOI:** 10.1155/2013/638563

**Published:** 2013-05-14

**Authors:** Andrés Ortiz, Antonio A. Palacio, Juan M. Górriz, Javier Ramírez, Diego Salas-González

**Affiliations:** ^1^Communications Engineering Department, University of Malaga, 29004 Malaga, Spain; ^2^Department of Signal Theory, Communications and Networking, University of Granada, 18060 Granada, Spain

## Abstract

Current medical imaging systems provide excellent spatial resolution, high tissue contrast, and up to 65535 intensity levels. Thus, image processing techniques which aim to exploit the information contained in the images are necessary for using these images in computer-aided diagnosis (CAD) systems. Image segmentation may be defined as the process of parcelling the image to delimit different neuroanatomical tissues present on the brain. In this paper we propose a segmentation technique using 3D statistical features extracted from the volume image. In addition, the presented method is based on unsupervised vector quantization and fuzzy clustering techniques and does not use any a priori information. The resulting fuzzy segmentation method addresses the problem of partial volume effect (PVE) and has been assessed using real brain images from the Internet Brain Image Repository (IBSR).

## 1. Introduction

Recent advances in the medical imaging systems make it possible to acquire high resolution images with high tissue contrast. Moreover, these systems provide images up to 16-bit depth, corresponding to 65535 intensity levels. On the other hand, the human vision system is not able to recognize more than several tens of gray levels. Thus, image processing techniques are necessary to exploit the information contained in medical images, to be successfully used in CAD systems. In addition, computer-aided tools can analyze the volume image in a reasonable amount of time. These are valuable tools for diagnosing some neurological disorders such as schizophrenia, multiple sclerosis, the Alzheimer's [1] disease, or other types of dementia. Image segmentation consists in parcelling or delimiting the image into different regions according to some properties or features describing these regions. In brain magnetic resonance imaging (MRI), segmentation consists in delimiting neuroanatomical tissues present on a healthy brain: white matter (WM), gray matter (GM), and cerebrospinal fluid (CSF). All of the nonrecognized tissues or fluids may be classified as suspected of being pathological. Segmentation process can be addressed in two ways. While the first consists in manual delineation of the structures usually performed by experts, the latter aims to use automatic or semiautomatic techniques which use statistical features that describe different regions on the image. Some of these techniques use the image histogram to define different tissues by applying a threshold, under the assumption that a tissue is characterized by an intensity level or by intensity levels within an interval [2, 3]. In the ideal case, three different image intensities should be found in the histogram corresponding to GM, WM, and CSF, assuming the resolution to be high enough to ensure that each voxel represents a single tissue type. Nevertheless, variations in the contrast of the same tissue are found in an image due to RF noise or shading effects caused by magnetic field variations. These variations which affect the tissue homogeneity on the image are a source of errors for automatic segmentation methods. Other approaches model the intensity histogram as probability distributions [4–6] or by a set of model vectors computed by vector quantization techniques [7, 8], reducing the segmentation problem to model the peaks and valleys present in the histogram. There are other histogram-based segmentation approaches that take into account the relative position of the peaks and valleys or other statistics extracted from the histogram [8–10]. However, histogram-based techniques usually does not take into account the spatial information contained in the image, and different images may have similar histogram profiles. On the other hand, segmentation has been addressed in other works by means of contour detection techniques [11, 12], region-based techniques [13], or other approaches that seek for voxels belonging to an initial class following specific geometrical models [14]. Segmentation may also be addressed as a classification task, which can be accomplished by supervised or unsupervised learning. Clustering techniques group similar voxels in an unsupervised way, according to a similarity criterion [15], while statistical classifiers may use the expectation-maximization (EM) algorithm [12, 16, 17], maximum likelihood estimation (ML), or markov random fields [18]. In addition, fuzzy variants of the *k-means* algorithm have also been widely used as they avoid abrupt transitions in the classification process [19] and address the PVE issue (i.e., voxels can contain signal from several tissues at the same time due to limited image resolution). In this paper we propose a segmentation method based on first and second order statistical features extracted from the image. There are segmentation approaches for 2D-MRI data. However, as MRIs are 3D in nature, we use 3D statistical features extracted from overlapped cubes moving through the image to accomplish a 3D segmentation approach. Moreover, local and nonlocal statistical descriptors extracted from the image are modelled in an unsupervised way using a self-organizing map, computing a reduced number of prototypes representing all the voxels in the image. In addition, the degree a voxel is modelled by an SOM prototype is computed by means of clustering the SOM units using the FCM algorithm [20]. After this introduction, the rest of the paper is organized as follows.  Section 2 presents the database used to evaluate our proposal and introduces the image preprocessing stage and the main techniques used such as SOM and FCM for modeling data and clustering the SOM, respectively.  Section 3 shows the segmentation approach proposed in this paper and the results obtained using the images from the IBSR database are depicted in Section 4. Finally, conclusions are drawn in Section 5.

## 2. Materials and Methods

**Figure 1:**
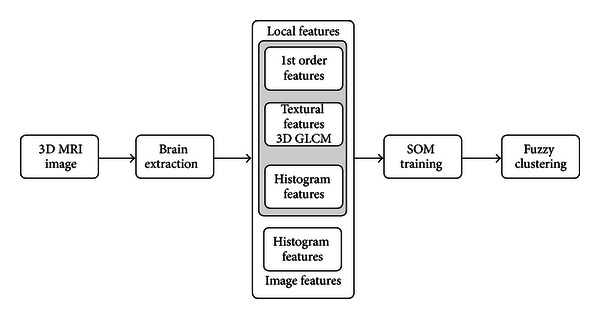
Block diagram of the segmentation method.

This section consists of six subsections which explain in detail the segmentation method presented in this paper and summarized in the block diagram shown in Figure 1. Moreover, the databases used to evaluate the performance of the proposes algorithm and the metric applied for quantitative assessment of the results are also provided in the following subsections.

### 2.1. Databases

The performance of our proposal has been evaluated in comparison with other methods, using the Internet Brain Segmentation Repository (IBSR) from the Massachusetts General Hospital [23]. This database provides 18 T1-weighted volumetric images, of 256 × 256 × 128 voxels, with voxels dimensions between 0.84 × 0.84 × 1.5 mm^3^ and 1 × 1 × 1.5 mm^3^, corresponding to subjects with ages between 7 and 71 years. The images are spatially normalized into the Talairach orientation and processed by the Center for Morphometric Analysis (CMA) at Massachusetts General Hospital with the biasfield *autoseg* routines for nonuniformity correction. In addition, IBSR database also provides manual segmentation references performed by expert radiologists. These segmented images were used as a reference to test our approach as usual in other works which use the IBSR 2.0 database [21, 22].

### 2.2. Image Preprocessing

Although the images are already registered in the database, they contain nonbrain structures such as scalp and skull. These structures have to be removed before dealing with segmentation. In our case, nonbrain structures were removed using the BET 2.0 tool [24] from FSL package [25], running two iterations on every subject. We run BET twice on each subject since nonbrain material remains on the MRI after one iteration and three iterations tend to remove some parts of the brain. Thus, running BET twice was determined to be optimum. Figure 1 shows the entire process, from original images to segmented ones.

### 2.3. Feature Extraction

**Figure 2:**
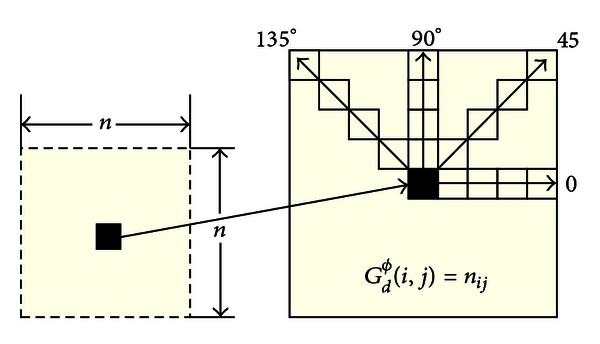
2D gray level cooccurrence matrix (GLCM) computation. Main directions (0°, 45°, 90°, and 135°) are used and average value is associated with central voxel.

Unlike other MRI segmentation approaches which use 2D statistical descriptors [22], we used 3D statistical descriptors extracted from overlapping cubes slided across the volumetric image. A similar method to extract 3D features is applied in [26] for 3D computerized tomography (CT) images. These descriptors include first and second order features, as (local) histogram features computed from each cube. First order features are the intensity level of the central voxel and mean and variance of the intensity of the voxels in the window. Second order statistics aim to describe the texture of the image as they take into account the relationship among voxels in a window. This way, Haralick et al. [27] proposed 14 features computed using the gray level cooccurrence matrix (GLCM), which is a structure that describes the cooccurring intensity values at a given offset. In other words, the GLCM provides information on how often a graylevel occurs at different directions. Usually, four directions are considered in the 2D case: *ϕ* = 0°, *ϕ* = 45°, *ϕ* = 90°, and *ϕ* = 135°. However, Haralick suggests using the mean value of the features computed for the four directions to guarantee rotation invariance. Moreover, symmetric GLCM (i.e., taking into account voxels separated by −*d* and *d* voxels) is a common choice in image analysis [27]. The structure of the 2D-GLCM is shown in Figure 2, where *n*_*ij*_ is the number of cooccurrences of gray levels *i* and *j* at a distance *d* and a specific direction. Thus, the GLCM, matrix defined as *G*_*d*_^*ϕ*^(*i*, *j*), is a square matrix of size *N*, where *N* is the total number of voxels in the window, so that (*i*, *j*) entry represents the number of cooccurrences of gray levels *i* and *j* for voxels separated at a distance *d* in direction *ϕ*.

**Figure 3:**
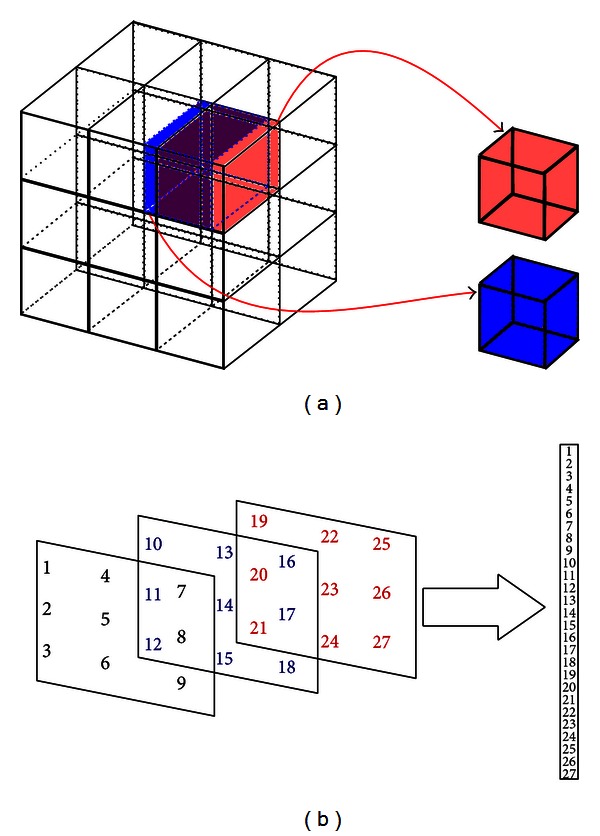
(a) 3D overlapped windows extraction process. Note that overlapping is shown as color mixture. (b) 3 × 3 × 3 cube vectorizing example. Image is split into slices depending on the *z* coordinate. Values in these slices correspond to the column vector index.

In the case of 3D-GLCM, cubes of size *w* × *w* × *w* instead of square windows (*w* × *w*) have to be considered. Moreover, the windowing process has been performed computing *w* slices in each window. The choice of the window size plays an important role in the classification process, as it may determine the discrimination capabilities of the extracted features. The use of small windows reduces the computational burden and keeps resolution but it may not be able to capture the texture. On the other hand, large windows will capture textural properties but they increase memory and processing requirements and may result in resolution loss. This way, we choose 3 × 3 × 3 windows as a trade-off between performance and resolution. This process is depicted in Figure 3.

While four independent directions exist in 2D for GLCM calculation, 13 independent directions are found in 3D, and GLCM computation can be generalized as

**Figure 4:**
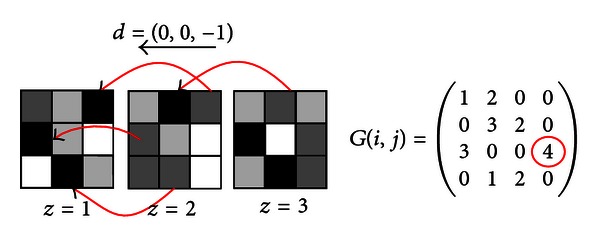
3D GLCM calculation in direction (0,0, −1). *z* values indicate different slices (*z coordinate*). Arrows show the relationship between voxels for computing the cooccurrence value in the direction indicated.

(1) Gdϕ(i,j) =∑z =1Vz− dz∑y =1Vy− dy∑x =1Vx− dx{1,if(Q(x,y,z)=i) ∧(Q(x+dx,y+dy,z+dz)   =j),0,otherwise,                      i,j=1,…,N,

where *N* is the number of gray levels present in the image or subimage considered for GLCM calculation, *V* = (*V*_*x*_, *V*_*y*_, *V*_*z*_) is the position of the voxel, and *d* = (*d*_*x*_, *d*_*y*_, *d*_*z*_) are the distances in each direction.  Figure 4 shows a calculation example of 3D GLCM for *d* = (0,0, −1) direction and *N* = 4 gray levels.

This way, 3D GLCM is computed through an offset *d* = (*d*_*x*_, *d*_*y*_, *d*_*z*_), where *d*_*x*_ and *d*_*y*_ correspond to 2D offset (as in 2D GLCM) and *d*_*z*_ indicates the *z* coordinate. As the offset can be applied in *x*, *y*, or *z* axes, there are 27 possible offset directions. However, as *G*_*d*_ = *G*_−*d*_^*T*^ there are only 13 independent directions as indicated in (1). This deals with 13 3D-GLCMs computed from each cube.

Regarding implementation details, cubes are vectorized as shown in Figure 3 to speed up the process in matlab [28]. Image vectorization aims to convert a 3D image into a matrix which contains a number of columns corresponding to the number of extracted cubes. Computations with these structures are considerably faster in matlab.

Once 3D GLCM has been defined, Haralick's textural features can be computed as in 2D, but using the 3D GLCM as previously defined in (1). Mathematical details on Haralick's textural features used in this work are provided in the appendix and can also be found in [8, 26, 27].

In addition to 3D Haralick features, we extract local histogram-based features from each 3D window. These features include maximum probability local intensity, mean, variance, skewness, entropy, energy and kurtosis [15].

Moreover, intensity probability in terms of the entire image is also included in the feature set. Thus, the entire feature set extracted from the image is summarized in Table 2.

Features computed from each window (cube) are extracted from the image and associated with the central voxel which is described by 23 features (i.e., feature space is composed by *23-dimensional* feature vectors).

### 2.4. Background in SOM

The self-organizing map [29] is a well-known bioinspired clustering algorithm which aims to discover the most representative and most economic representation of data and its relationships [29, 30]. SOM consists of a number of units arranged in a two- or three-dimensional lattice, and each of them stores a prototype vector. During the training stage, the prototypes retain the most representative part of the input data, while the units on the output space holding similar prototypes (in terms of euclidean distance) are moved closer together. In other words, units on the output space are close as their prototypes are similar, and units being apart on the output space hold different prototypes. Thus, some important features of the input space can be inferred from the output space [30].

1. Input space modelling: the prototypes computed during the SOM training, *ω*_*i*_, provide an approximation to the input space, as each prototype models a part of it.
2. Topological order: units on the output map are arranged into a 2D or 3D lattice, and their position depends on the specific features of the input space.
3. Density distribution: SOM reveals statistical variations on the distribution of the input data. This way, a higher density on the output space corresponds to a higher density on the input space.
4. Feature selection: prototypes computed from the input data space represent the data manifold. Thus, the algorithm reduces the input space to a set of prototype vectors.

The process mentioned previously is performed in a competitive way, where only one neuron wins (i.e., its prototype vector is the most similar to the input data instance) with each input data instance. Nevertheless, prototypes of neurons belonging to the neighbourhood of the wining unit (called best matching unit (BMU)) are also updated. Let the SOM units be linearly indexed. The BMU *ω*_*i*_ is computed as

(2) $$\begin{array}{c} ||v_{k}-\omega_{i}||\leq ||v_{k}-\omega_{j}||\;\forall i\neq j\in S, \end{array}$$

where *S* = {1,…, *n*} is the output space for an SOM composed by *n* units and *v*_*k*_ is the *k*th input. Moreover, prototypes of units belonging to the neighbourhood of the wining unit (also called best matching unit (BMU)) are also updated according to

(3) $$\begin{array}{c} \omega_{j}\left(t+1\right)=\omega_{j}\left(t\right)+\alpha \left(t\right)h_{i,j}\left(t\right)\left(v_{k}-\omega_{j}\left(t\right)\right), \end{array}$$

where *α*(*t*) is the learning factor and *h*_*i*,*j*_(*t*) is the neighbourhood function defining the unit surrounding the BMU *ω*_*i*_. Both *α*(*t*) and *h*_*ij*_(*t*) decrease exponentially with *t*. Thus, the prototype vectors *ω*_*i*_ quantize the data manifold and represent the cluster center of the data mapped on each BMU.

### 2.5. SOM Clustering Using FCM

SOM can be seen as a clustering method as it quantizes the feature space by a number of prototypes, and each prototype can be considered as the most representative vector of a class. On the other hand, the prototypes are projected onto a two- or three-dimensional space while topology (i.e., distribution of SOM units in the projection space) is preserved. In that sense, SOM assumes that each map unit acts as a single cluster. In this work, input space is composed by feature vectors whose coordinates represent a different feature as presented in Section 2.3. Thus, each voxel is represented by a 23-dimensional vector and SOM is used to group these vectors (i.e., voxels) in different classes. This way, SOM performs *hard* clustering over the feature vectors which describe image voxels, and the cluster a voxel belongs to is represented by the BMU corresponding to its feature vector. In other words, SOM quantizes the feature space. However, this simple model that considers each unit as a different cluster does not exploit valuable information contained in SOMs and it is referred to its topological preservation property (i.e., nearby units in the map model similar data [29]). Thus, SOM provides extra advantages over classical clustering algorithms if more than one unit represents the same class, and a range of SOM units act as BMU for a subset of the data manifolds. This adds flexibility to the clustering algorithm and allows to compute a set of prototype vectors for the same class. Nevertheless, since each cluster can be prototyped by a set of model vectors, grouping SOM units is necessary to define cluster borders [8]. SOM clustering can be addressed by specific clustering algorithms such as CONN linkage [31, 32], which implements a hierarchical agglomerative technique using the topological information contained in the map and the relationships between the SOM layer and the data manifold to build clusters. Nevertheless, taking into account the membership probability of a voxel to a cluster requires fuzzy or probabilistic clustering techniques. Hence, we used fuzzy c-means (FCM) algorithm as a voxel can belong to more than one cluster at the same time according to a certain membership measure [20, 33, 34]. FCM for SOM can be formulated as follows. Let *v*_*k*_ be the *k* feature vector representing the *k* voxel of the image and *ω*_*i*_ the prototype associated with the *i*-unit on the SOM. An objective function can be defined as

(4) $$\begin{array}{c} J_{m}=\sum_{k\;=1}^{N}\;\sum_{i\;=1}^{C}u_{ki}{||v_{k}-\omega_{i}||}^{2},\;1\leq m\leq \infty , \end{array}$$

where *N* is the number of data samples (voxels), *C* is the number of SOM units, and *u*_*ij*_ is the membership function defined as

(5) $$\begin{array}{c} u_{ki}=\sum_{l\;=1}^{C}{\left(\frac{||v_{k}-c_{i}||}{||v_{k}-c_{l}||}\right)}^{-2/\left(m-1\right)}, \end{array}$$

where *c*_*l*_ is the center of cluster *l*, defined as

(6) $$\begin{array}{c} c_{l}=\frac{\sum_{k\;=1}^{N}u_{ki}^{m}v_{k}}{\sum_{k\;=1}^{N}u_{ki}^{m}}. \end{array}$$

Fuzzy clustering is carried out by optimizing the objective function (4) until

(7) $$\begin{array}{c} \underset{ki}{\max}\left\{\left|u_{ki}^{t+1}-u_{ki}^{t}\right|\right\}<\epsilon , \end{array}$$

where *ϵ* ∈ [0,1] and *t* represents the iteration steps. This iterative procedure will converge to a local minimum of *J*_*m*_ [33]. 

**Figure 5:**
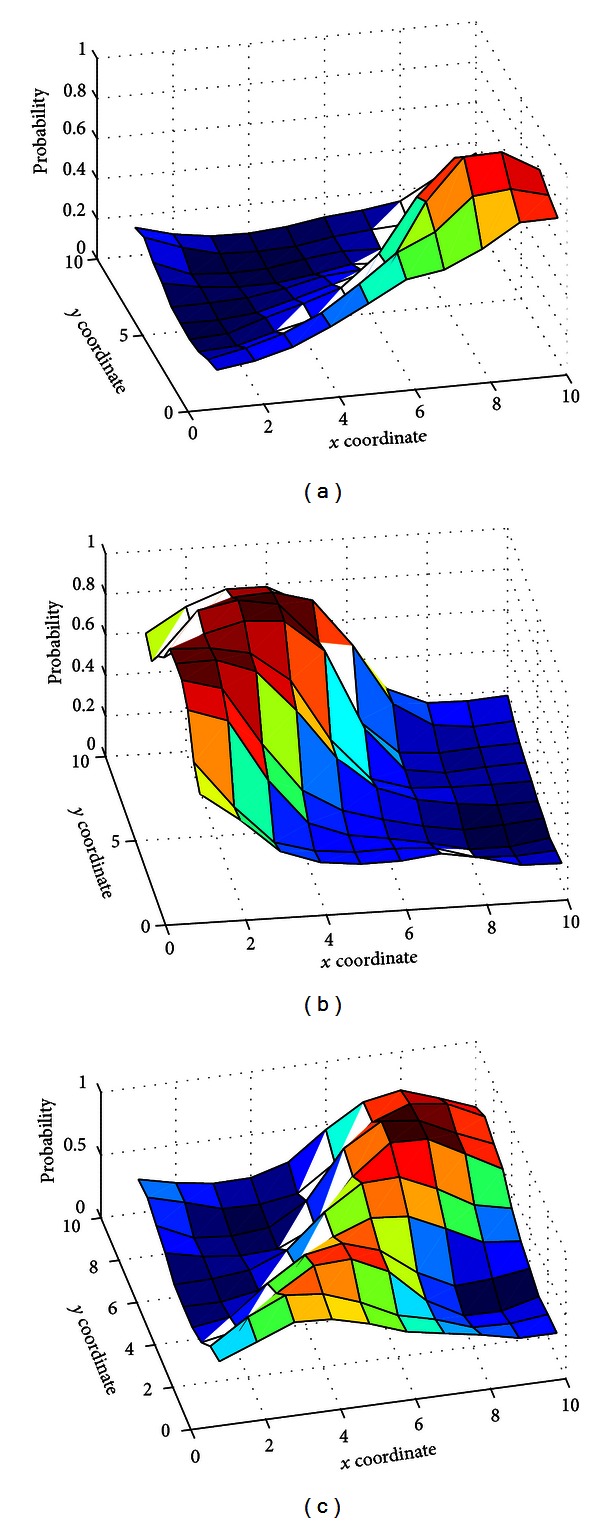
SOM units membership probability for WM (a), GM (b), and CSF (c) clusters according to FCM clustering for IBSR volume 7.

**Figure 6:**
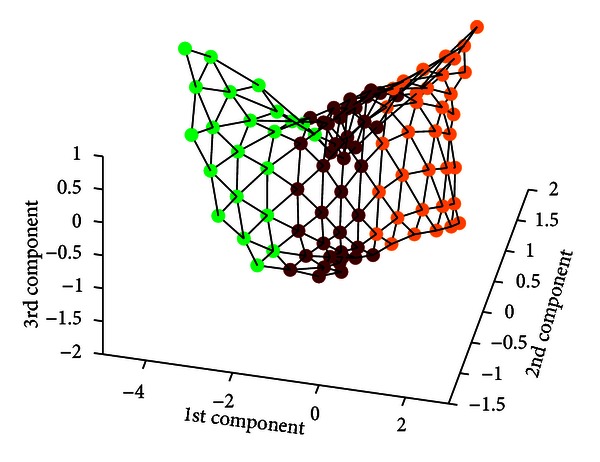
3D projection of the SOM prototypes for IBSR volume 7. Units have been colored according to the *maximum membership* criterion. Maroon, orange, and green represent WM, GM, and CSF prototypes, respectively.

Once the SOM is trained and clustered, each voxel (described by its corresponding feature vector) is mapped to a cluster, so that it belongs to a specific tissue with a probability.  Figure 5 shows the membership value assigned to each SOM unit for the three clusters (WM, GM, and CSF), and Figure 6 shows the projection of the SOM prototypes in 3D where the units have been colored according to the *maximum membership* criterion using the probabilities computed by FCM clustering. However, this method deals with hard clustering and does not take into account partial volume effect (PVE) [22, 35, 36]. In order to take into account PVE in our implementation, we introduced a *thresholding* parameter *τ* to determine whether a BMU is included in two classes at the same time. Thus, we define the *D* matrix as

(8) $$\begin{array}{c} d_{ik}=\left|u_{ik}-u_{jk}\right|,\;\;j=1,\ldots ,C,\;\;i\neq j. \end{array}$$

Thus, voxels whose BMU fulfills the *d*_*ik*_ < *τ* constraint are included in *i* and *k* clusters (i.e., belong to *i* and *k* tissues).

In order to identify the tissue corresponding to each cluster, we use the fact that GM voxels usually present lower intensity values than CSF, and WM voxels present the higher intensity values due to the MRI acquisition process. This way, the cluster with the lower mean intensity value is associated with GM voxels and the cluster with the higher mean intensity values is associated with WM voxels.

### 2.6. Feature Selection

As shown in Section 2.3, first order and second order (textural) and histogram-based features are extracted from each cube. However, using all the features does not provide the best results as they may not be discriminative enough for the three tissues. Moreover, using non-discriminative features can deteriorate the clustering results. Thus, a feature selection stage was performed using a subset of 5 training images to compute the most discriminative features. This is addressed by a genetic algorithm (GA), using the parameters described in [8] which evolves an initial population of solutions (permutations of the features) aiming to minimize the fitness function defined as

(9) $$\begin{array}{c} \text{fitness}=-\left(J_{\text{WM}}+J_{\text{GM}}+J_{\text{CSF}}\right), \end{array}$$

where *J*_*T*_ is the mean *Jaccard* coefficient [7, 15, 23] for a subset of 5 images (volumes no. 7, no. 8, no. 9, no. 10, and no. 11). It measures the average overlap between the segmented image and the segmentation reference provided by the database. This metric is used in many works [21–23, 37] to assess the performance of segmentation algorithms and can be defined as

(10) $$\begin{array}{c} J\left(S_{1},S_{2}\right)=\frac{\left|S_{1}\cap S_{2}\right|}{\left|S_{1}\cup S_{2}\right|}, \end{array}$$

where |·| represents the cardinality of the set.

**Figure 7:**
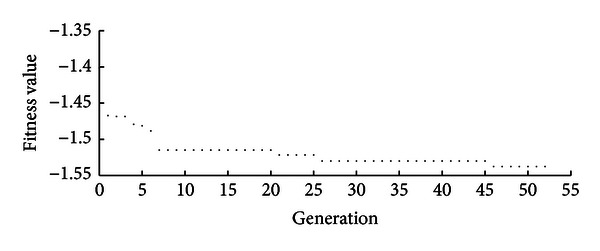
Fitness function in the optimization process. Convergence is achieved in 50 generations.

Figure 7 shows the fitness value for 60 generations, which are enough for the GA to converge. The optimized feature set is shown in Table 3.

Thus, these features have been used to process the images on the IBSR database and to provide the segmentation outcomes based on the Jaccard index as shown in Section 4.

### 2.7. SOM Topology Selection

**Figure 8:**
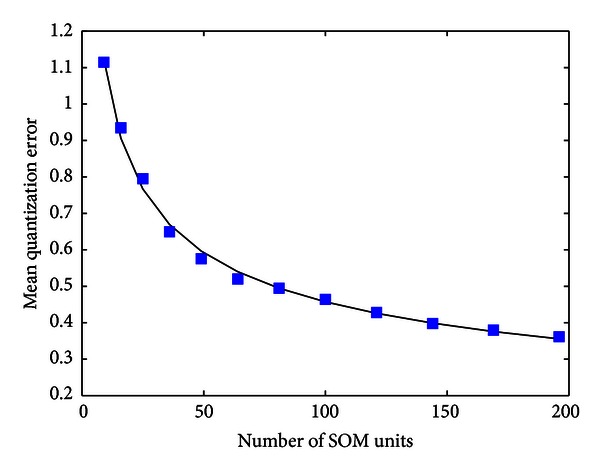
Mean quantization error as a function of the number of SOM units.

The number of SOM units usually determines the performance of the clustering.  Figure 8 shows the quantization error as a function of the number of units used in the model, computed using the equation

(11) $$\begin{array}{c} Q_{\mathrm{err}}=\frac{\sum_{i\;=1}^{N}||v_{i}-\omega_{\text{bmu}}^{i}||}{N}, \end{array}$$

where *ω*_bmu_^*i*^ is the BMU corresponding to data sample *v*_*i*_ and *N* is the number of data samples in the dataset.

The quantization error in this figure represents a measure of the reconstruction error, which tends to stabilize from 64 units. Thus, we choose 10 × 10 units map as a trade-off between quantization error and performance. Moreover, 3D SOM layer with hexagonal lattice was used as it yields better segmentation results.

## 3. Segmentation Procedure

Unsupervised segmentation using SOM requires to cluster the SOM units after training. This can be addressed using a standard clustering algorithm or a specific algorithm developed to cluster the SOM layer, as shown in Section 2.5. The overall process is summarized in Algorithm 1.

Cluster assignment of SOM units can be addressed in two ways. Since FCM computes membership probability, the units can be assigned to the cluster providing the maximum probability. This method uses the *maximum membership* criterion and assumes that a voxel only belongs to a specific cluster and does not address the partial volume effect (PVE) (i.e., a voxel can contain signal from different tissues due to the limited resolution of the acquisition process). The second approach can assign a voxel to different clusters simultaneously if the membership probability is above a predefined threshold *τ*. These two previous approaches have been implemented in the experiments conducted and experimental results are provided with and without PVE correction. *τ* = 0.02 has been used in the experiments performed in Section 4, meaning that voxels whose membership probability differs in *τ* for two different clusters are assigned to these two clusters (tissues) simultaneously.

## 4. Experimental Results

**Figure 9:**
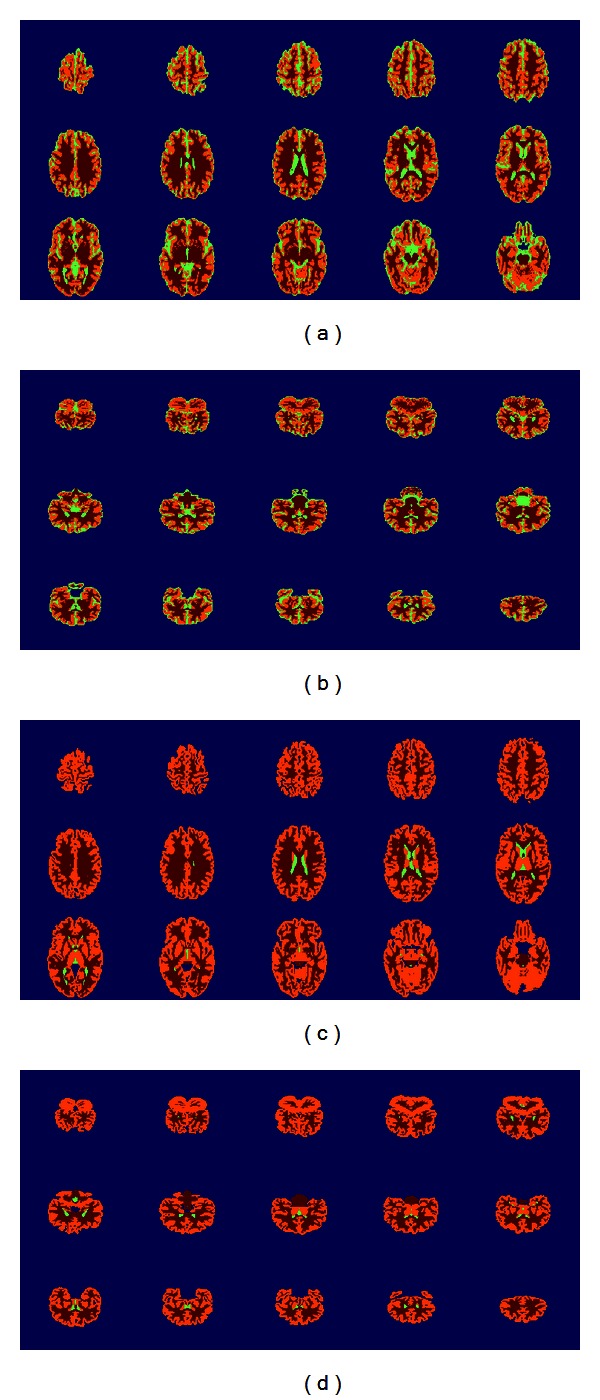
Segmentation results for the IBSR volume 7. Some slices of the axial and coronal planes are shown in (a) and (b), respectively. Segmentation performed by experts is shown in (c) and (d) for the axial and coronal planes, respectively.

Numerous experiments were carried out to assess the performance of the proposed algorithm using the IBRS2 database, as it provides real brain MRIs.  Figure 9 shows the segmentation results for some slices of axial and coronal planes, respectively, for the IBSR volume 7 using the *maximum membership* criterion to *defuzzify* the clustering result. In this case, no PVE correction is applied as each voxel only belongs to a single cluster.

Thus, in Figure 9 each tissue is shown as a different color. Specifically, CSF, GM, and WM are shown as green, orange, and maroon to identify them in the same figure. In addition, Figures 9(c) and 9(d) show the segmentation references from the IBSR database (i.e., manual segmentation by expert radiologist or *ground truth*). It is worth noting that expert segmentations included in the IBSR database does not include internal CSF spaces. However, our approach also delineates sulcal CSF. This is the main source of difference between our segmentation outcomes and the ground truth. Thus, Jaccard index for CSF is not as high as in the WM or GM case.

**Figure 10:**
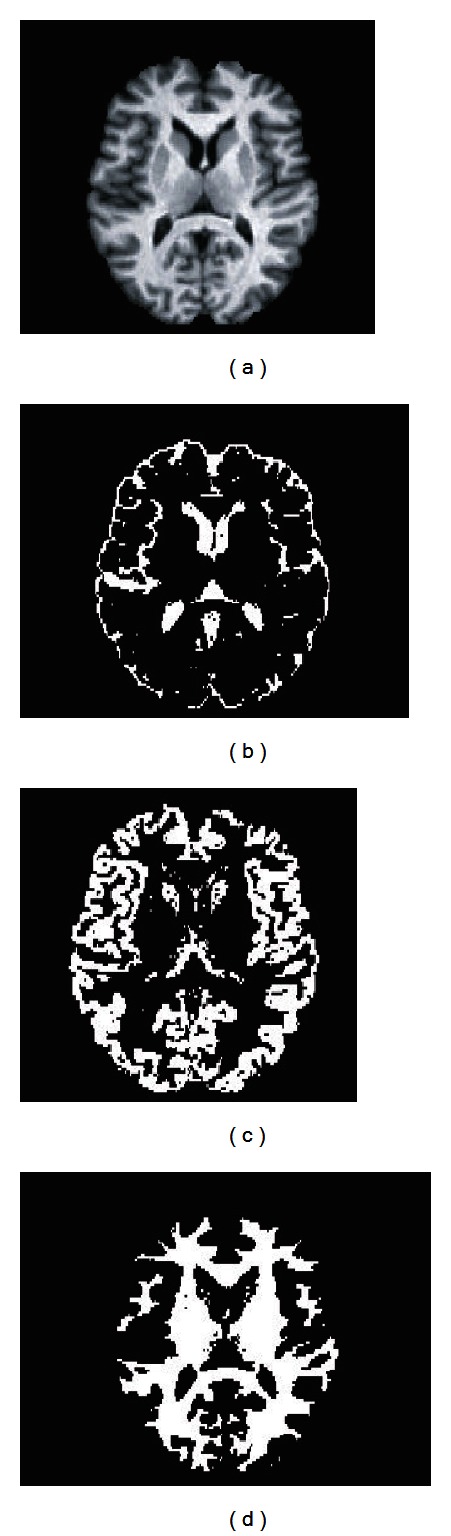
Axial slice from volume no. 7 (a). Segmentation results show CSF (b), WM (c), and GM (d).

In order to show segmentation outcomes, Figure 10 presents a slice of the original IBSR2 volume no. 7 after brain extraction and the segmented tissues. In this figure, CSF, GM, and WM are shown in Figures 10(b), 10(c), and 10(d), respectively. Coronal plane is shown in Figure 12.

**Figure 11:**
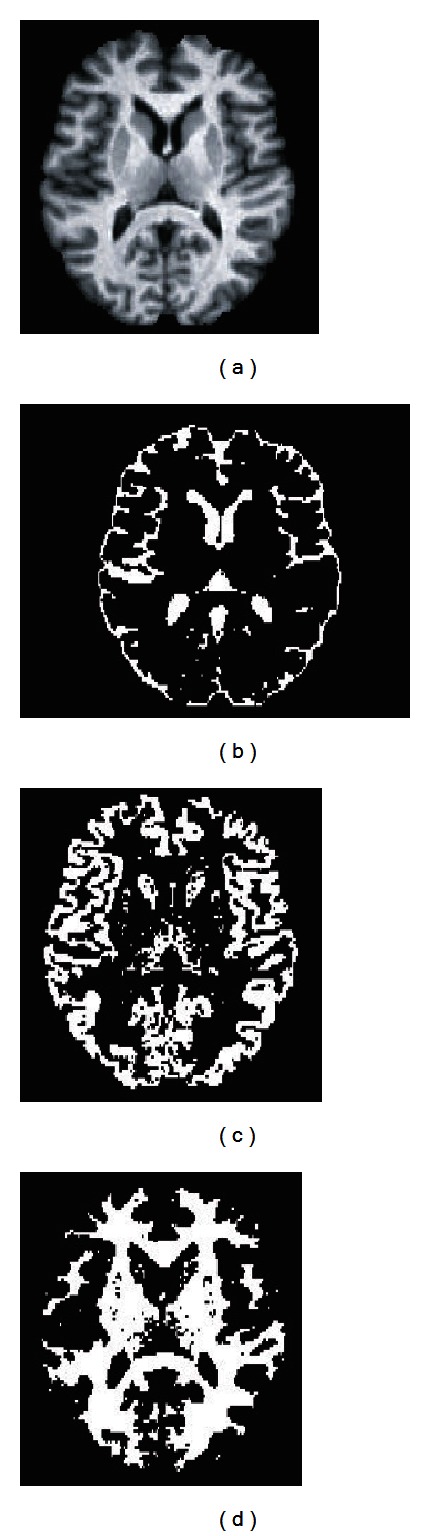
Axial slice from volume no. 7 (a). Segmentation results show CSF (b), WM (c), and GM (d) with PVE correction, *τ* = 0.02.

**Figure 12:**
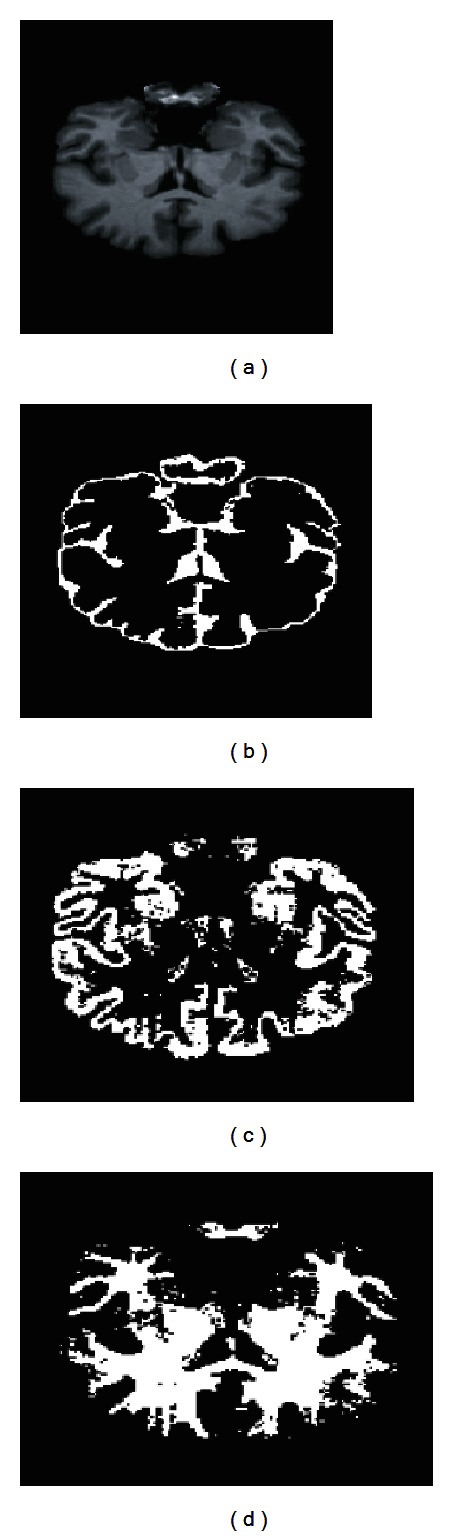
Coronal slice from volume no. 7 (a). Segmentation results show CSF (b), WM (c), and GM (d).

**Figure 13:**
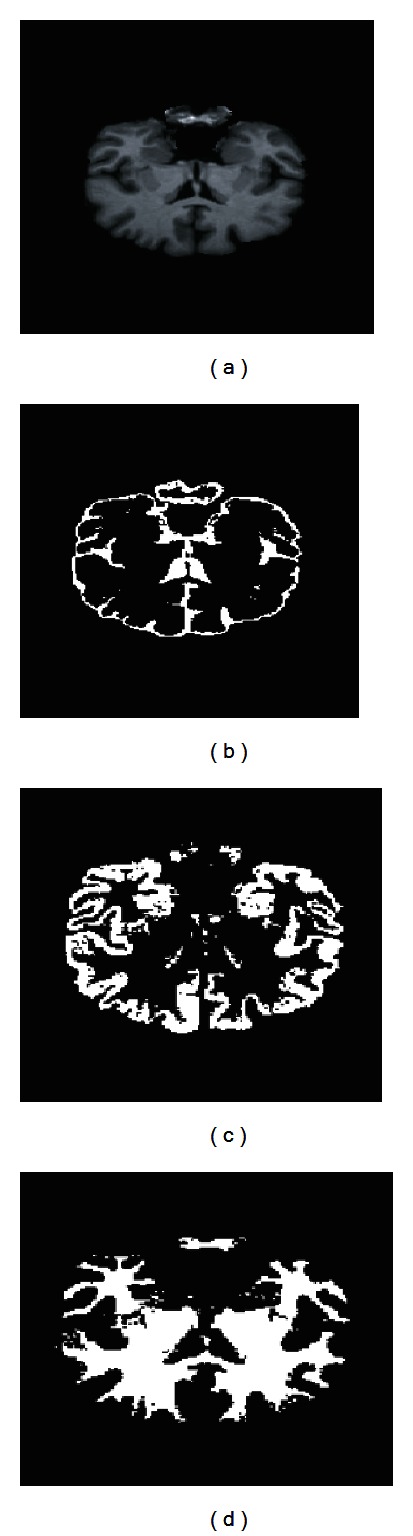
Coronal slice from volume no. 7 (a). Segmentation results show CSF (b), WM (c), and GM (d) with PVE correction, *τ* = 0.02.

**Figure 14:**
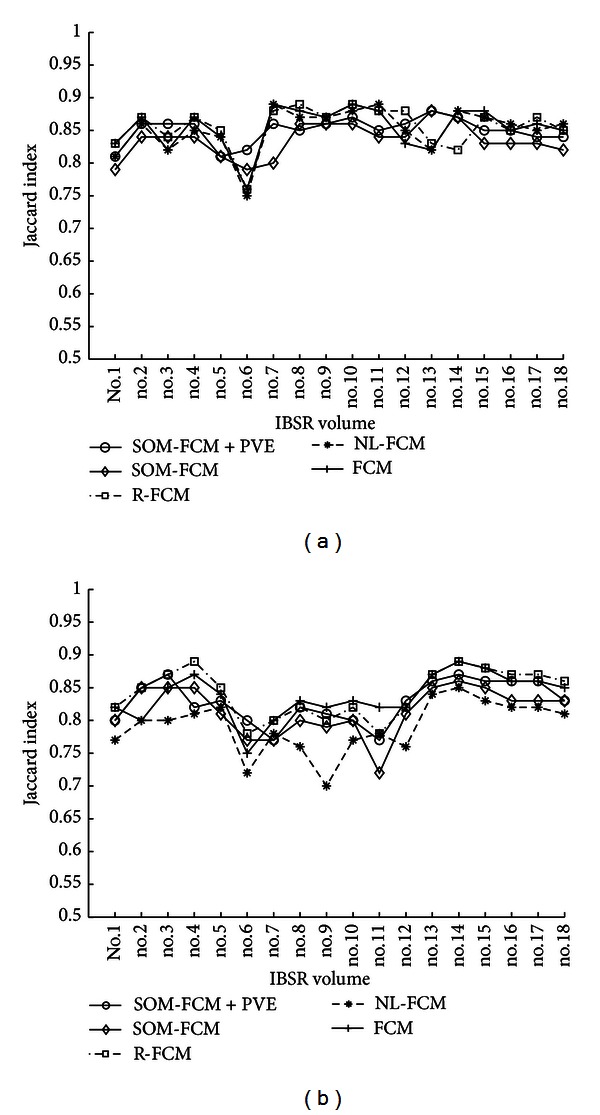
Jaccard index calculated throughout the images in the IBSR2 database.

In addition, PVE correction is applied as explained in Section 2.5, and the results are presented in Figures 11, 13, and 14. As shown in these figures, PVE correction improves the results, specially for GM delineation.  Figure 14 presents the Jaccard index for the 18 volumes on the IBSR2 database. Moreover, a comparison with other segmentation techniques is presented for comparison. As shown in these figures, our method outperforms the FCM method applied over the voxel intensity levels and also performs better for some volumes than other methods combining fuzzy clustering and intensity inhomogeneity compensation techniques. Our method tends to better delineate WM and also delineates correctly the CSF. Moreover, brain extraction stage may cause differences in the final segmentation results in terms of the Jaccard index, as the number of voxels in the segmentation references may differ depending on the brain extraction technique (i.e., brain extraction by manual delineation).

## 5. Conclusions

In this paper, we present MRI segmentation methods using 3D statistical features (Tables 1 and 4). These features include first and second order statistics computed using overlapping cubes moving across the image. In addition, local histogram features computed from each cube are used to compose the feature space. The feature vectors associated with each nonbackground voxel are unsupervisedly modelled by an SOM, reducing the feature space to a number of prototypes each of them representing a set of voxels. These prototypes are grouped to define the cluster borders in the SOM layer using FCM, allowing a specific prototype to model voxels belonging to different tissues simultaneously. This way, PVE correction is incorporated to the segmentation algorithm. The algorithm has been assessed by experiments regarding the modelling capabilities using the proposed feature set and the proposed clustering technique combining SOM and FCM. Moreover, a GA-based feature selection stage is used over a subset of images to compute the most discriminative features. Segmentation results using the selected features show improvements over other segmentation methods depending on the specific volume and clearly outperform FCM. In addition, results using *maximum membership* criterion to *defuzzify* the clustering result and fuzzy clusters (i.e., it is possible to assign a voxel to two tissues at the same time) are shown. The first approach does not correct PVE while the latter does. Segmentation techniques could help to find causes of brain disorders such as Alzheimer's disease (AD). In fact, the segmentation algorithm presented in this paper is part of a larger study performed by the authors on the tissue distribution for neurological disorders characterization and the early diagnosis of AD.

## Figures and Tables

**Algorithm 1:**
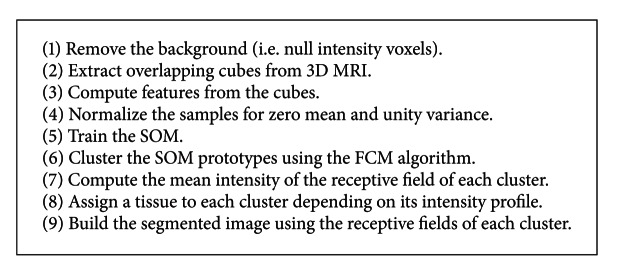
SOM-FCM segmentation algorithm for 3D MRI.

**Table 1:** 3D directions in spherical coordinates and offset coding. Offset (*d*) indicates radius value.

Direction (*ϕ*, *θ*)	Offset vector	Direction (*ϕ*, *θ*)	Offset vector
(0,0)	(0, *d*, 0)	(90,45)	(−*d*, 0, −*d*)
(45,0)	(−*d*, *d*, 0)	(90,135)	(*d*, 0, −*d*)
(90,0)	(−*d*, 0,0)	(45,45)	(−*d*, *d*, −*d*)
(135,0)	(−*d*, −*d*, 0)	(45,135)	(*d*, −*d*, −*d*)
(0,45)	(0, *d*, −*d*)	(135,45)	(−*d*, −*d*, −*d*)
(0,90)	(0,0, *d*)	(135,135)	(*d*, *d*, −*d*)
(0,135)	(0, −*d*, −*d*)		

**Table 2:** Feature set extracted from 3D image.

Index	Feature	Index	Feature
3D Haralick (Textural)			

1	Energy	7	Sum average
2	Entropy	8	Dissimilarity
3	Correlation	9	Cluster shade
4	Contrast	10	Cluster tendency
5	Homogeneity	11	Maximum probability
6	Variance	12	Difference variance

Local histogram			

13	Central voxel Intensity		
14	Intensity mean		
15	Intensity variance		

Local histogram			

16	Mean intensity	20	Skewness
17	Intensity variance	21	Kurtosis
18	Energy	22	Maximum probability intensity
19	Entropy		

Image histogram			

23	Intensity probability		

**Table 3:** Optimized feature set.

Index	Feature type	Feature
1	3D-GLCM/Haralick-textural	Energy
4	3D-GLCM/Haralick-textural	Contrast
6	3D-GLCM/Haralick-textural	Homogeneity
9	3D-GLCM/Haralick-textural	Cluster shade
12	3D-GLCM/Haralick-textural	Inverse variance
13	1st order	Voxel intensity
14	1st order	Voxel mean intensity
15	1st order	Voxel intensity variance
18	Local histogram	Energy
20	Local histogram	Skewness
21	Local histogram	Kurtosis

**Table 4:** Mean and standard deviation of the Jaccard index for the segmentation methods in Figure 14.

Algorithm	Ref.	WM index	GM index
SOM-FCM + PVE (*τ* = 0.02)	—	0.85 ± 0.02	0.83 ± 0.03
SOM-FCM	—	0.83 ± 0.02	0.82 ± 0.02
NL-FCM	[21]	0.85 ± 0.04	0.79 ± 0.04
R-FCM	[22]	0.85 ± 0.04	0.84 ± 0.04
FCM	[22]	0.83 ± 0.03	0.82 ± 0.03

## Appendix

### Haralick's Textural Features Computed Using the 3D GLCM

Energy:

(A.1) $$\begin{array}{c} \sum_{i\;=1}^{N}\sum_{j\;=1}^{N}G_{d}^{\phi }{\left(i,j\right)}^{2}. \end{array}$$

Entropy:

(A.2) $$\begin{array}{c} \sum_{i\;=1}^{N}\sum_{j\;=1}^{N}G_{d}^{\phi }\left(i,j\right)\log_{2}\left(G_{d}^{\phi }\left(i,j\right)\right). \end{array}$$

Correlation:

(A.3) $$\begin{array}{c} \sum_{i\;=1}^{N}\sum_{j\;=1}^{N}G_{d}^{\phi }\left(i,j\right)\frac{\left(i-\mu_{x}\right)\left(i-\mu_{y}\right)}{\sigma_{x}\sigma_{y}}. \end{array}$$

Contrast:

(A.4) $$\begin{array}{c} \sum_{n\;=1}^{N}\left(n^{2}\sum_{i\;=1}^{N}\sum_{j\;=1}^{N}G_{d}^{\phi }\left(i,j\right)\right),\;n=\left|i-j\right|. \end{array}$$

Inverse difference moment (ASM, homogeneity):

(A.5) $$\begin{array}{c} \sum_{i\;=1}^{N}\sum_{j\;=1}^{N}\frac{1}{1+{\left(i-j\right)}^{2}}G_{d}^{\phi }\left(i,j\right). \end{array}$$

Variance:

(A.6) $$\begin{array}{c} \sum_{i,j}^{N}{\left(1-\mu \right)}^{2}G_{d}^{\phi }\left(i,j\right). \end{array}$$

Sum average:

(A.7) $$\begin{array}{c} \sum_{i\;=1}^{2N-1}\left(ip_{x+y}\left(i\right)\right). \end{array}$$

Dissimilarity:

(A.8) $$\begin{array}{c} \sum_{i\;=1}^{N}\sum_{j\;=1}^{N}\left(i-j\right)G_{d}^{\phi }\left(i,j\right). \end{array}$$

Cluster shade:

(A.9) $$\begin{array}{c} \sum_{i\;=1}^{N}\sum_{j\;=1}^{N}{\left(i+j-\mu_{x}-\mu_{y}\right)}^{3}G_{d}^{\phi }\left(i,j\right). \end{array}$$

Cluster prominence:

(A.10) $$\begin{array}{c} \sum_{i\;=1}^{N}\sum_{j\;=1}^{N}{\left(i+j-\mu_{x}-\mu_{y}\right)}^{4}G_{d}^{\phi }\left(i,j\right). \end{array}$$

Maximum probability:

(A.11) $$\begin{array}{c} \sum_{i\;=1}^{N}\sum_{j\;=1}^{N}\underset{i,j}{\max}\left\{G_{d}^{\phi }\left(i,j\right)\right\}. \end{array}$$

Difference variance:

(A.12) $$\begin{array}{c} \sum_{i\;=1}^{N}\sum_{j\;=1}^{N}i^{2}p_{x-y}\left(i\right). \end{array}$$

*Notations*

- *N* is the number of distinct gray levels in the window.
- *G*(*i*, *j*) corresponds to the (*i*, *j*)th entry in the 3D GLCM.
- MDM = ∑_*i*,*j*_^*N*^ *G*_*d*_^*ϕ*^(*i*, *j*) marginal-probability distribution matrix.
- *p*_*x*_(*i*) = ∑_*i*=1_^*N*^ *G*_*d*_^*ϕ*^; *p*_*y*_(*j*) = ∑_*i*=1_^*N*^ *G*_*d*_^*ϕ*^ *i*th and *j*th entry in the marginal-probability distribution matrix, respectively.
- *μ*_*x*_, *μ*_*y*_, *σ*_*x*_, *σ*_*y*_, are, respectively, the means and standard deviations of the partial probability density functions *p*_*x*_ and *p*_*y*_. In the case of variance calculation, *μ* represents the mean of the values within the 3D GLCM. Symmetrical GLCM yields *μ*_*x*_ = *μ*_*y*_ and *σ*_*x*_ = *σ*_*y*_.
- *σ*_*x*_ = (∑_*i*=1_^*N*^∑_*j*=1_^*N*^(*i*−*μ*_*x*_)^2^)^1/2^; *σ*_*y*_ = (∑_*i*=1_^*N*^∑_*j*=1_^*N*^(*j* − *μ*_*y*_)^2^)^1/2^.
- *μ*_*x*_ = ∑_*i*=1_^*N*^∑_*j*=1_^*N*^ *iG*_*d*_^*ϕ*^(*i*, *j*); *μ*_*y*_ = ∑_*i*=1_^*N*^∑_*j*=1_^*N*^ *jG*_*d*_^*ϕ*^(*i*, *j*).
- *p*_*x*+*y*_(*k*) = ∑_*i*=1_^*N*^∑_*j*=1_^*N*^ *G*_*d*_^*ϕ*^(*i*, *j*),  *i* + *j* = *k*,  *k* = 2,3,…, 2*N*.
- *p*_*x*−*y*_(*k*) = ∑_*i*=1_^*N*^∑_*j*=1_^*N*^ *G*_*d*_^*ϕ*^(*i*, *j*),  |*i* − *j* | = *k*,  *k* = 1,2,…, *N*.
